# Macrophage-fibroblast interplay: a target for neuropeptide-based treatment of fibrotic disease?

**DOI:** 10.1186/1479-5876-10-S3-P22

**Published:** 2012-11-28

**Authors:** Sita Virakul, Willem A Dik, Leo J Hofland, P Martin van Hagen, Virgil ASH Dalm

**Affiliations:** 1 Dept. of Immunology, Erasmus MC, Rotterdam, The Netherlands; 2 Dept. of Internal Medicine, Erasmus MC, Rotterdam, The Netherlands

## Background

Fibrosis is defined as an excessive accumulation of extracellular matrix (ECM) components with an associated loss of normal tissue architecture and function. Fibrosis occurs in many different auto-inflammatory (auto-immune) diseases. Current therapies targeting fibrosis are limited and we are in great need for novel therapeutic regimens.

In fibrosis, abnormal fibroblast proliferation and ECM production by fibroblasts is stimulated by factors released by different cell types, amongst which macrophages. Macrophages produce many pro-fibrotic factors, such as IL-6, CCL2, PDGF and TGF-beta.

In rodent models of rheumatoid arthritis and inflammatory bowel disease, somatostatin analogues exerted strong anti-inflammatory effects, by inhibition of pro-inflammatory cytokine secretion. Somatostatin analogues bind to somatostatin receptors (sst) and we demonstrated sst subtype 2 expression on human macrophages.

In the present study we investigated the effects of somatostatin, octreotide, a clinically used somatostatin analogue, and cortistatin, an endogenously expressed somatostatin-like peptide, on macrophage-induced fibroblast proliferation.

## Materials and methods

A human fetal lung fibroblast cell line (HFL1) was used in this study. Monocytes were isolated from healthy donors and allowed to differentiate into macrophages. Fibroblasts were co-cultured with macrophages or incubated with conditioned macrophage media in the presence or absence of lipopolysaccharide, somatostatin, octreotide or cortistatin. Thymidine incorporation was used to measure fibroblast proliferation.

## Results

Culture of fibroblasts with media of unstimulated and LPS stimulated macrophages resulted in an approximately 2-fold and 3-fold increase in proliferation, respectively.

Conditioned media from octreotide-treated LPS-activated macrophages showed a statistically lower activation of fibroblast proliferation. In co-culture experiments somatostatin, octreotide and cortistatin significantly inhibited unstimulated macrophage-induced fibroblast proliferation by approximately 30%, whereas these compounds significantly inhibited LPS-stimulated macrophage-induced fibroblast proliferation by 20%. As octreotide does not bind to sst_1_ expressed on fibroblasts, at least the effects of octreotide on fibroblast proliferation should be mediated via macrophages.

## Conclusions

In conclusion, macrophages significantly stimulate human fetal lung fibroblast proliferation *in vitro*. Treatment of activated macrophages with somatostatin analogues significantly decreased proliferation of fibroblasts. Ongoing studies will reveal the cytokines and growth factors involved in these actions, and whether their release by macrophages is affected by somatostatin analogues. Based on these preliminary findings somatostatin analogues may be promising agents in treatment of fibrotic diseases.

